# *α*-Synuclein E46K Mutation and Involvement of Oxidative Stress in a *Drosophila* Model of Parkinson's Disease

**DOI:** 10.1155/2021/6621507

**Published:** 2021-07-03

**Authors:** Samaneh Reiszadeh Jahromi, S. R. Ramesh, David I. Finkelstein, Mohammad Haddadi

**Affiliations:** ^1^Department of Biology, Faculty of Science, University of Sistan and Baluchestan, Zahedan, Iran; ^2^Department of Studies in Zoology, University of Mysore, Manasagangothri, Mysore, India; ^3^Parkinson's Disease Laboratory, Florey Institute of Neuroscience and Mental Health, 30 Royal Parade, University of Melbourne, Parkville, Australia; ^4^Department of Biology, Faculty of Science, University of Zabol, Zabol, Iran

## Abstract

Parkinson's disease (PD) is an age-associated neurodegenerative condition in which some genetic variants are known to increase disease susceptibility on interaction with environmental factors inducing oxidative stress. Different mutations in the *SNCA* gene are reported as the major genetic contributors to PD. E46K mutation pathogenicity has not been investigated as intensive as other *SNCA* gene mutations including *A30P* and *A53T*. In this study, based on the GAL4-UAS binary genetic tool, transgenic *Drosophila melanogaster* flies expressing wild-type and E46K-mutated copies of the human *SNCA* gene were constructed. Western blotting, immunohistochemical analysis, and light and confocal microscopy of flies' brains were undertaken along with the survival rate measurement, locomotor function assay, and ethanol and paraquat (PQ) tolerance to study *α*-synuclein neurotoxicity. Biochemical bioassays were carried out to investigate the activity of antioxidant enzymes and alterations in levels of oxidative markers following damages induced by human *α*-synuclein to the neurons of the transgenic flies. Overexpression of human *α*-synuclein in the central nervous system of these transgenic flies led to disorganized ommatidia structures and loss of dopaminergic neurons. E46K *α*-synuclein caused remarkable climbing defects, reduced survivorship, higher ethanol sensitivity, and increased PQ-mediated mortality. A noticeable decline in activity of catalase and superoxide dismutase enzymes besides considerable increase in the levels of lipid peroxidation and reactive oxygen species was observed in head capsule homogenates of *α*-synuclein-expressing flies, which indicates obvious involvement of oxidative stress as a causal factor in *SNCA*^*E46K*^ neurotoxicity. In all the investigations, *E46K* copy of the *SNCA* gene was found to impose more severe defects when compared to wild-type *SNCA*. It can be concluded that the constructed *Drosophila* models developed PD-like symptoms that facilitate comparative studies of molecular and cellular pathways implicated in the pathogenicity of different *α*-synuclein mutations.

## 1. Introduction

*SNCA* gene is mapped to human chromosome 4q22.1 encoding *α*-synuclein polypeptide. It was recognized as the primary causative gene in the etiology of Parkinson's disease (PD) [1]. Prior to its association with PD, *α*-synuclein was characterized as a presynaptic protein acting at neuron terminals in the rat brain [2]. A human homologue of *SNCA* was discovered in 1993 as the contributor to amyloid plaques deposition in Alzheimer's disease (AD) [3]; the first familial case of parkinsonism was described in 1997, showing a dominant inheritance pattern for an *SNCA* mutation (*A53T*) [4]. It was indicated that *α*-synuclein is a constituent of the Lewy body (LB), showing how *SNCA* is completely linked to PD [5]. Since then, duplication/triplications and a number of *SNCA* missense mutations viz., *A53T*, *A30P*, *E46K*, *Q51D*, and *H50Q* have been associated with parkinsonism [6]. All these mutations are uncommon and have variable penetrance [7]. Misfolded proteins and aggregations are typical features of neurodegenerative disorders. The Lewy body (LB) is the key pathologic characteristic of PD involving *α*-synuclein fibrils as a constituent. Importantly, mutations in *SNCA* are sufficient to provoke familial PD, with autosomal dominant inheritance pattern.

*α*-Synuclein can acquire several diverse biochemical forms including membrane-bound alpha helix, *ß*-sheet oligomers, unfolded monomers, as well as insoluble LB fibrils [8]. Mutated *α*-synuclein variants such as *A53T*, *A30P*, and *E46K* show a tendency to aggregate more rapidly than wild-type *SNCA* [9, 10].

Oxidative stress (OS) is a putative causal agent in the neurodegenerative process of PD [11]. Auto-oxidation of dopamine and the subsequent neuromelanin production in a multifaceted pathway generate reactive oxygen species (ROS) and OS, resulting in dopaminergic neuron failure in PD patients [12–14]. Furthermore, higher iron levels in dopaminergic neurons have been contributed to free radical production through the Fenton and Harber–Weiss reaction triggering neurodegeneration process [15]. *α*-Synuclein, dopamine, and iron appear to accelerate these processes [16, 17].

*Drosophila melanogaster* is a widely known model organism to explore the biology of many biological processes including those occurring in PD [18]. Mohite et al. [19] have studied *G51D*, *H50Q*, and *E46K* mutations of the *SNCA* gene in *Drosophila* and showed shortened lifespan and age-dependent climbing defects in the transgenic flies. They have concluded that continued *α*-synuclein oligomer formation is the major cause of dopaminergic neuron death. Sakai et al. [20] have employed transgenic flies to report accelerated *α*-synuclein monomer protein formation and degradation resistance in *SNCA*^*E46K*^ mutants compared to wild-type and other mutations associated with *SNCA* gene in familial PD. These studies try to elucidate the molecular basis of *SNCA* mutation neurotoxicity. However, the involvement of OS in *SNCA*^*E46K*^ pathogenicity remains elusive. In order to investigate the neurotoxicity of the *E46K* mutation of the *SNCA* gene and the implication of OS, a transgenic *UAS-hSNCA*^*E46K*^*Drosophila* line was constructed. Lifespan, locomotor function, brain histology, biochemical bioassay, ethanol vapour exposure, and paraquat (PQ) induced mortality carried out on adult male flies following *E46K α*-synuclein overexpression to highlight the possible causative role of OS.

## 2. Materials and Methods

### 2.1. Fly Stocks

#### 2.1.1. Human *SNCA* DNA Preparation and Cloning

Random integration of P-element transgenesis was applied in order to construct a transgenic *Drosophila* line expressing human *SNCA* [21]. *SNCA* cDNA plasmid (Sino Biological Inc., China) was subcloned into a pUAST vector [22]. The *SNCA*^*E46K*^, pMT 18T-*SNCA* vector served as the template for site-directed mutagenesis. DNA segments were sequenced to verify sequence accuracy. Inserts were cloned in the pUAST vector, which was used to establish transgenic lines (C-CAMP Transgenic Fly Facility, NCBS, Bangalore, India). A total of 5 lines were achieved for the wild-type and mutated *SNCA* gene. Neuronal expression of the target gene was induced by *elav-Gal4.* Of all the constructed *UAS-hSNCA*^*E46K*^ transgenic fly stocks, the one with the expression level equal to the wild-type *SNCA* was identified using quantitative Western blot analysis. Both the selected *UAS-SNCA*^*E46K*^ and *UAS-SNCA*^*WT*^ stocks showed insertion on the second chromosome.

Bloomington *Drosophila* stocks were used in the current study. *Ddc-Gal4* (# 7010) to induce gene expression in dopaminergic neurons, *elav*^*C155*^*-Gal4* (# 458) for Pan neural expression, along with *GMR-Gal4* (# 9146) to overexpress *hSNCA* in *Drosophila* eye structure. *UAS-mCD8::GFP* line (# 32186) was employed to visualize neuron morphology. *w*^1118^ (# 5905) was used as the control genotype. All *Drosophila* stocks were raised and kept at 22 ± 1°C and relative humidity of 70–80% on standard wheat cream agar media, containing dry yeast granules in a 12 h light/12 h dark cycle in a vivarium. All the study assays were conducted on adult male flies.

### 2.2. Western Blotting

This assay was carried out based on the method of Bolt and Mahoney [23]. Total head homogenates of 25 flies of each group underwent a standard protein extraction process. Following SDS-PAGE, the gel was transferred to the blotting unit, and after protein bands transfer onto the nitrocellulose membrane, the blot was blocked with 3% BSA at 4°C overnight. Then the blot was kept for incubation with 1:5000 anti-human *α*-synuclein mouse IgG monoclonal antibody (Cat # ABH0261, Invitrogen, USA), at 4°C overnight. The blot was washed with 0.3% phosphate buffer saline Triton X (PBSTx) for the duration of 30 min. Next, the blot was incubated with 1:1,000 diluted buffer of secondary rabbit anti-mouse HRP conjugated antibody (Cat # 6728, Abcam, UK) in 1X PBS for 1 h at room temperature. The blot was given a wash and then developed using a chromogenic substrate (1X TMB) to detect the horseradish peroxidase (HRP). The expression of the test protein can be monitored by the development of bluish-green color bands to which secondary antibody is bound. Anti-*ß*-actin antibody (Cat # 8224, Abcam, UK) was utilized as a loading control.

### 2.3. Scanning Electron Microscopy (SEM)

SEM was conducted to investigate the surface morphology of flies' eyes using the method of Kimmel et al. [24]. Adult flies were anesthetized and preserved in 25% ethanol at room temperature. The samples were dehydrated in 50%, 75%, and 2 × 100% ethanol for 24 h at each step and dried using hexamethyldisilazane (HMDS). Then to allow HMDS to evaporate, uncapped vials were kept under a fume hood overnight. Dried samples were examined with a Carl Zeiss EVO LS-10 SEM. The captured micrographs were analyzed by the FLEYE tool [25, 26] in ImageJ software [27] to demonstrate the level of ommatidia irregularity. PPi represents the probability of a certain stage of irregularity in a given ommatidium. The index can be a value between 0 and 4 where 1 stands for complete regularity and 4 accounts for total irregularity. A total of 5 samples of each genotype were examined.

### 2.4. *Drosophila* Eye Preparation for Ultramicrotomy

Preparation of adult *Drosophila* eye for thin sectioning was performed as per our previous study [28]. Heads of flies were fixed in 2.5% glutaraldehyde: 2% paraformaldehyde at 4°C overnight. Osmium tetroxide, 0.5%, was used as the secondary fixative. Next, samples were washed and dehydrated by serial ethanol treatment and subsequent two washing stages with propylene oxide. The infiltration was achieved by storing test tissues in a solution mixture of fresh epoxy resin as well as propylene oxide while holding on a shaker at room temperature. The samples were embedded in moulds filled with freshly prepared epoxy resin. Then the blocks were incubated at 60°C for 48 hours. Finally, the blocks were cut into sections of 1 *μ*m thickness using Leica EM UC6 ultramicrotome (Leica Mikrosysteme, Austria). Semithin sections underwent toluidine blue staining and were observed with a light microscope. The captured images were analyzed by ImageJ software [26]. A total of 5 samples of each genotype were examined.

### 2.5. Confocal Microscopy

To inspect and assess CNS dopaminergic neurons in *E46K* transgenic *Drosophila* flies, brain dissection was carried out on the ice and subsequently handled for fixation using cold 4% paraformaldehyde for the duration of 30 min. Then, PBSTx was used to wash brain samples for 40 min, with 4 changes per 10 min using a shaker at room temperature. At last, samples were mounted using VECTASHIELD mounting medium (Vector Laboratories, USA). The prepared samples were examined by confocal microscope (LSM 710, Carl Zeiss, Germany) and analyzed by ImageJ software [27]. A total of 5 samples of each genotype were examined.

### 2.6. Longevity Assay

To assess the *SNCA* neurotoxicity survival rate of flies, lately enclosed adult flies were clustered in groups of 20. The flies were transferred to fresh media in 3-day intervals. The number of dead flies was quantified in each turnover, and alive insects were considered for calculation of survival [28].

### 2.7. Negative Geotaxis Assay

The locomotor activity of the flies was characterized by negative geotaxis assay as formerly described [29]. Ten adult male flies were placed in a plastic tube that was marked at 8 cm from the base. Flies were kept for 10 min rest to acclimatize to the new environment. Next, the flies were lightly tapped down to the base. The number of flies that climbed past the 8 cm mark on the tube in 10 seconds was quantified. Six biological replicates of each genotype were analyzed, and the corresponding values were represented as mean ± standard error.

### 2.8. Locomotion Tracing Assay

To trace the locomotion behaviour of the flies, the iFLY system was used [30]; this system analyzes the flies' movement in an automated fashion. The iFLY uses a single digital camera to track the trajectories of up to 20 individual flies. Briefly, 10 flies were transferred to a glass tube and then placed in a chamber. To facilitate 3-D tracking, two mirrors were placed at equal angles at the back of the tube to allow side images capture by the camera. A 10-min rest was given to the flies for acclimatizing to the new place. Next, flies were gently tapped down every 30 seconds and their locomotion was recorded for 90 seconds. The captured video clips were analyzed by C-Trax software [30]. The movement of each individual fly was quantified, and corresponding graphs were plotted by the software. Six biological replicates of each genotype were analyzed.

### 2.9. Ethanol Exposure

Hyperactivity and sedation are of main behavioural responses to acute ethanol exposure that are conserved from flies to mammals [31]. Excessive ethanol intake is associated with ROS generation and reduction in endogenous antioxidant defenses [32]; therefore, the ethanol exposure test was undertaken to investigate locomotion dysfunction in *SNCA*^*E46K*^ PD model flies. The standard protocol of Maples and Rothenfluh [33] was used to measure the ethanol sensitivity of the flies. For this assay, eight flies were collected one day before ethanol exposure. The ST50 (the time required to get half of the flies stationary) and RC50 (the time required for complete recovery of half of the sedated flies) were measured and expressed in minutes. ST50 and RC50 values of eight biological replicates of each genotype were averaged and represented as mean ± standard error.

### 2.10. Paraquat (PQ) Treatment and Mortality Assay

PQ, a commercial herbicide, is a neurotoxic agent capable of inducing PD-like symptoms in mammalian and *Drosophila* models [34]. PQ inhibits mitochondrial complex I and leads to excess ROS production [35] that finally results in neuronal death. PQ administration to flies was carried out in a treatment chamber [34]. The PQ chambers were prepared by insertion of a round piece of filter paper presoaked with 5% sucrose and air-dried at the bottom of a glass vial. Then the paper was soaked with 100 *μ*l of PQ (15 mM). The experimental flies were starved for 3 h at 25°C and then transferred to the PQ treatment chamber for 48 h. Fresh filter papers were provided every 24 h, and the mortality rate at 24 h was recorded. The lethal concentration of PQ causing 50% mortality (LC50) was determined by regression analysis, and a sublethal concentration of PQ (15 mM) was applied to investigate survivorship of the *SNCA*^*E46K*^ transgenic flies. Eight replicates per every genotype were analyzed, and the results are reported.

### 2.11. Biochemical Bioassays

The samples for biochemical assays were homogenates of heads of 100 flies suspended in ice-cold phosphate buffer (PB) and centrifuged at 3,000 *g* for 10 min at 4°C. The supernatant was subsequently utilized to assess the activity of antioxidant enzymes and levels of oxidative markers. Three different homogenates were prepared from each genotype and served as biological replicates. Each biological replicate was divided into 3 microtubes and used in every bioassay.

As we previously described [36], the catalase (CAT) activity was assessed based on the H_2_O_2_ hydrolysis rate [37]. The enzyme activity was expressed as *μ*M of H_2_O_2_ used/min/mg protein. Superoxide dismutase (SOD) activity was measured based on SOD-mediated inhibition of pyrogallol auto-oxidation [38]. The enzyme activity was shown as units, wherein 1 unit corresponds to 50% inhibition of pyrogallol auto-oxidation. Reduced glutathione content was assessed according to the method of Hissin and Hilf [39] using O-phthalaldehyde (OPA). The glutathione level was estimated using a standard curve and was represented as *μ*g·GSH/mg protein. ROS levels were measured based on the fluorometric method with the DCFH-DA probe [40]. If ROS are present in the reaction, the nonfluorescent DCFH-DA probe gets quickly oxidized into 2′,7′-dichlorofluorescein (DCF), a highly fluorescent agent that can be spotted using fluorometric measurements. The DCF concentration in test samples was calculated using DCFH-DA standard curve. Lipid peroxidation (LPO) was assessed as per Ohkawa et al. [41] using thiobarbituric acid (TBA). The biochemical basis of LPO assay is malondialdehyde (MDA) synthesis, which is an end product of the LPO process which reacts with TBA and forms a chromogenic solution. The MDA contents of samples were measured using a tetramethoxypropane molar extinction coefficient (*ε*) value which is equal to 15,600 M^−1^·cm^−1,^ the. Estimation of total protein content was performed according to Lowry et al. [42]. The method is based on the reaction of peptide nitrogen and the copper ions in alkaline conditions and consequent Folin–Ciocalteu reagent reduction through copper-catalyzed oxidation of aromatic acids. The protein content of each sample was assessed using the bovine serum albumin (BSA) standard curve.

### 2.12. Statistical Analysis

Data were presented as mean ± SE. Kolmogorov–Smirnov and Shapiro–Wilk tests were applied to all the data series, and results indicate that all the experimental data follow the normal distribution. Therefore, mean comparison between groups was carried out by parametric tests including independent *t*-test and one-way ANOVA followed by Dunnett's post hoc test in SPSS (predictive analytics software) version 22.0. A *p* value of 0.05 was assumed as the minimum level of significance. Three significance level was considered, each represented by different asterisks numbers as demonstrated in all illustrations (^*∗*^*p* < 0.05, ^∗∗^*p* < 0.01, and ^∗∗∗^*p* < 0.001).

## 3. Results

**Figure 1:**
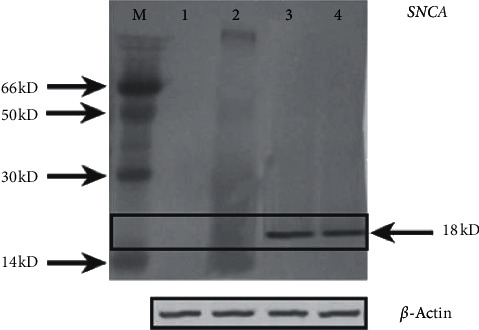
Human *SNCA* expression in the brain of transgenic flies. Western blot analyses of the human *α*-synuclein protein were made on the brain lysates of the flies expressing human *SNCA* gene via pUAST-*GAL4*-mediated gene expression. Lane description: marker (M), negative control genotype, *w*^1118^ (1), *UAS-E46K/+* (2), *SNCA*^*wt*^*/+; elav/+* (3), *SNCA*^*E46K*^*/+; elav/+* (4). The human *α*-synuclein protein was detected using anti-human *α*-synuclein mouse IgG monoclonal antibody (Invitrogen, USA). An anti-*Drosophila ß*-actin antibody (Abcam) was used as a control. Gel analysis tool in ImageJ software revealed no significant difference between bands in lanes 3 and 4 (*p*=0.057). This indicated equal expression of wild-type and *E46K* mutant copy of *SNCA* gene in the brain of the transgenic flies.

In the present study, *E46K α*-synuclein transgenic *Drosophila* stock was constructed, and *Gal4*-mediated *α*-synuclein gene expression in transgenic flies' brains was verified at the protein level. Western blot analysis on “*E46K; elav*” transgenic flies head samples was carried out. The results confirmed successful *Gal4*-UAS-mediated overexpression of *E46K* mutant form of the human *α*-synuclein gene in transgenic flies (Figure 1).

Using a gel analysis tool in ImageJ software [30], the respective bands of each genotype were quantified, and a mutant strain showing expression level with no significant difference to wild-type *α*-synuclein transgenic (*p*=0.057) strain was selected for further experiments. Following confirmation of *E46K α*-synuclein protein expression, behavioural, morphological, and biochemical analysis was performed.

**Figure 2:**
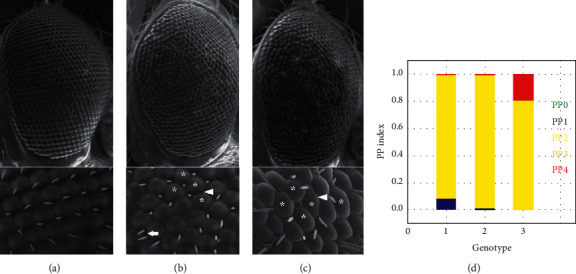
Overexpression of the *E46K* mutant human *α*-synuclein gene leads to the irregular surface morphology of the eye structures in transgenic flies. Scanning electron micrographs of ommatidial structures in *GMR-Gal4::UAS-SNCA*^*E46K*^ flies show degeneration of retinal neurons which is evident by the irregular organization of ommatidia, extra and/or absence of bristles. (a) *GMR/+*, (b) *GMR/SNCA*^*WT*^, and (c) *GMR/SNCA*^*E46K*^. ^*∗*^indicate an irregular shape of ommatidium; the arrow represents the absence of bristle; and the arrowhead represents extra bristles. The ImageJ FLEYE tool illustrates a higher rate of ommatidial irregularity in *SNCA*^*E46K*^ expressing flies (d) that accounts for more severe neuronal degeneration in *SNCA*^*E46K*^ expressing flies compared to that seen in the *SNCA*^*WT*^ (PPi = probability parameter for irregularity). Scale bar, 20 *μ*m. Five samples of each genotype were analyzed.

Preliminary evaluation of Drosophila eye structure was carried out in 10-day-old F1 progeny with a stereo microscope. Subsequent SEM studies in these transgenic lines showed that *E46K α*-synuclein gene overexpression results in degeneration of retinal neurons, a process that was obvious by observing irregular eye surface morphology (Figure 2).

**Figure 3:**
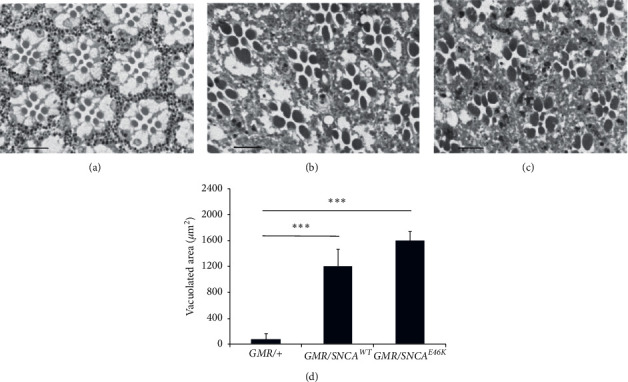
Retinal neuron degeneration in human *SNCA* transgenic flies. Photomicrographs of apical tangential sections of ommatidia in *E46K α*-synuclein transgenic flies show disorganized photoreceptor cells, vacuoles, and neurodegeneration (1000x magnification, toluidine blue-stained). (a) *GMR/+*, (b) *GMR/SNCA*^*WT*^, and (c) *GMR/SNCA*^*E46K*^. (d) Measuring total vacuolated area approves that degree of degeneration in *SNCA*^*E46K*^ flies is remarkably higher than observed in the *SNCA*^*WT*^ genotype (^*∗∗∗*^*p* < 0.001; by *t*-test; *n* = 15). Scale bar, 10 *μ*m.

Light microscopy analysis of the apical tangential sections of the fly ommatidia (Figure 3) revealed disorganized photoreceptor cells within the ommatidial structure and a higher level of neurodegeneration, evident by the observation of vacuoles in *E46K α*-synuclein transgenic flies.

**Figure 4:**
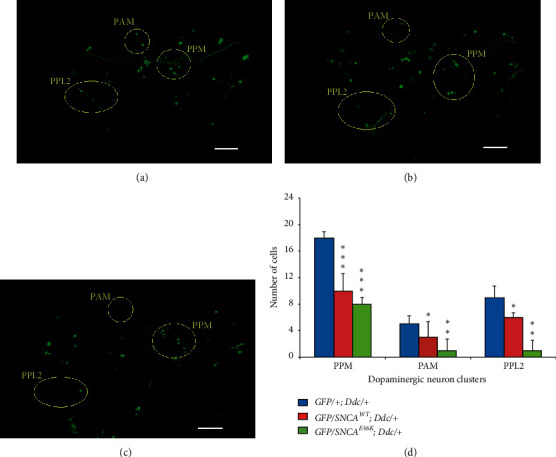
Confocal images of the brain of *E46K α*-synuclein transgenic flies demonstrate degeneration in protocerebral antero-medial (PAM), protocerebral postero-medial (PPM), and protocerebral postero-lateral (PPL) clusters of dopaminergic neurons. (a) *GFP/+; Ddc/+*, (b) *GFP/SNCA*^*WT*^*; Ddc/+*, and (c) *GFP/SNCA*^*E46K*^*; Ddc/+.* Scale bar, 50 *μ*m. (d) Cell counting indicates a significant reduction in the number of dopaminergic neurons up on *SNCA*^*E46K*^ overexpression, which was prominent for the PPM cluster (^*∗*^*p* < 0.05, ^*∗∗*^*p* < 0.01, ^*∗∗∗*^*p* < 0.001; by *t*-test; *n* = 15).

The *Ddc*-*Gal4* expression pattern was further assessed using the *Ddc*-GFP reporter gene complex. Confocal images of the brain of *E46K α*-synuclein transgenic flies reveal neurodegeneration in protocerebral antero medial (PAM), protocerebral posterior medial (PPM), as well as protocerebral posterior lateral (PPL) clusters of dopaminergic neurons (Figure 4).

**Figure 5:**
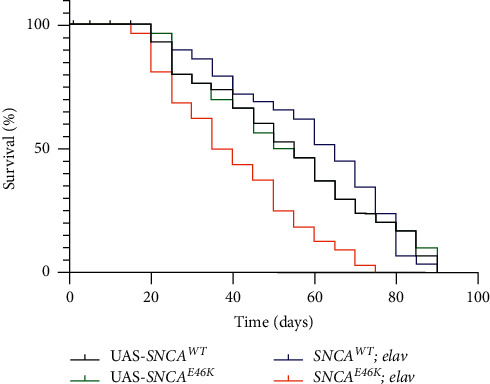
*SNCA* ^*E46K*^ expressing PD model flies live shorter lives. 360 one-day-old adult flies of each genotype were assayed for longevity. Statistical analysis using the Mantel–Cox log rank test revealed a significant decline in the maximum and median lifespan of the transgenic insects (*p* < 0.001).

Survival assay illustrates that PAN neuronal overexpression of *SNCA* led to reduced lifespan of the transgenic flies (Figure 5). The maximum lifespan of *UAS-SNCA*^*WT*^*/+; elav/+* flies was 85 ± 2 days while 70 ± 1 days for *UAS-SNCA*^*E46K*^*/+; elav/+* genotype. The median lifespan of *SNCA* expressing flies displayed a significant reduction when compared to the control genotype (*p* < 0.001; *n* = 1440).

**Figure 6:**
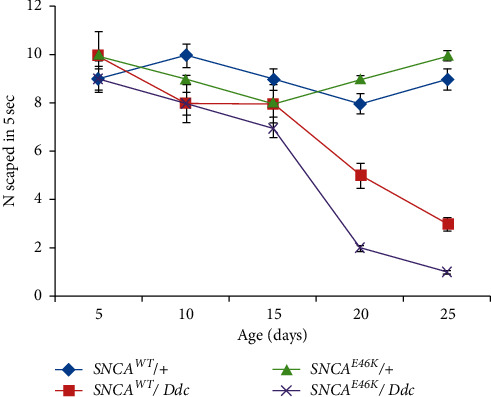
Decreased climbing ability of *E46K α*-synuclein transgenic flies in a negative geotaxis assay. Dopaminergic neuronal expression of *SNCA*^*E46K*^ in the brain of transgenic flies was driven by the promoter *Ddc-Gal4*. The descendants showed age-dependent defects in their climbing ability compared to the control genotype. Six replicates of each genotype were analyzed. The average number of flies crossing the 8 cm mark in 10 seconds is significantly decreased in 20-day-old and 25-day-old *E46K α*-synuclein expressing flies (*p* < 0.01; by one-way ANOVA; *n* = 24).

The negative geotaxis assay clearly showed that the climbing ability of transgenic flies in the negative geotaxis assay is diminished in an age-dependent manner as compared to control strains. Both the wild-type and *E46K* exacerbate the climbing potential of the flies at 20th and 25th day of their age. But the frequency of locomotion defects was higher in *E46K* mutant (Figure 6).

Based on the observed decrease in longevity and climbing defects, 20-day-old adult male flies were used for further behavioural and biochemical investigations.

**Figure 7:**
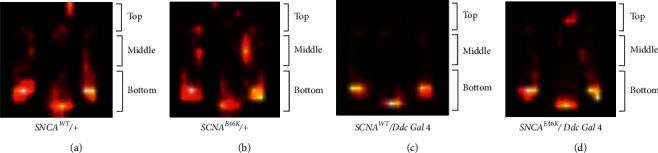
*SNCA* ^*E46K*^ transgenic flies move less and slower. An automated fly tracking using iFLY apparatus demonstrates the lower general movement of *SNCA*^*E46K*^*/+*; *Ddc/+* when compared to the control genotype *SNCA*^*E46K*^*/+*. The position of the control flies in the first 2,699 frames (a) is compared to the position of *SNCA*^*WT*^ expressing flies (b). The heat map figure shows a decline in locomotor activity among the PD model transgenic flies. Position histogram of 2,699 frames captured from control genotype, *SNCA*^*E46K*^*/+* (c), and that of *SNCA*^*E46K*^*/+*; *Ddc/+* genotype (d) indicates control flies spent more of their time on the top of the tube but are able to move across the whole tube. While *SNCA* transgenic *Drosophila* flies prefer to stay at the tube bottom and do not climb up. Lighter heat map spots indicate stationary objects.

Automated tracking of individual flies indicates a lower movement rate of *SNCA*^*E46K*^ model flies, and also, it was shown that these flies prefer to stay at the bottom of test tubes, while the control flies travel faster and climb up to the middle and top parts of the tube (Figure 7).

**Figure 8:**
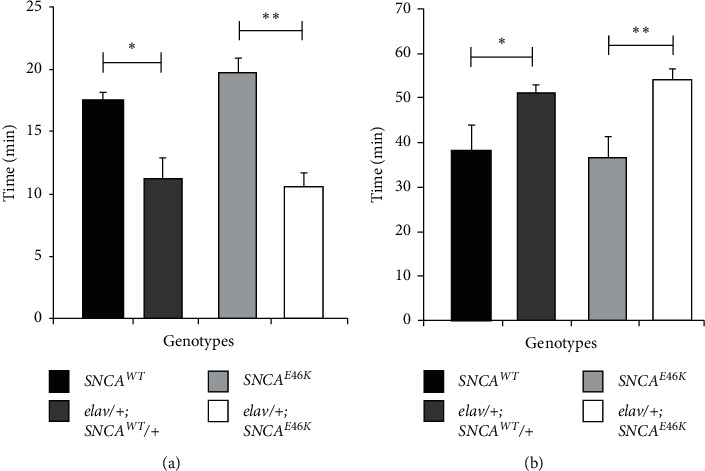
*SNCA* ^*E46K*^ PD model flies are more sensitive to ethanol vapour. Ethanol exposure sensitivity 50% (ST50) (a) and recovery 50% (RC50) (b) values of *SNCA*^*E46K*^ expressing transgenic flies were compared to the control genotype. The ST50 was 19.7 ± 2 min and 10 ± 1 min in the control genotype (*UAS-SNCA*^*E46K*^*/+*) and *elav; SNCA*^*E46K*^*/+* flies, respectively. The values show a significant decrease in the sensitivity of *SNCA*^*E46K*^ PD model flies (*p* < 0.01; by one-way ANOVA; *n* = 32). The recovery to 50% of the population was found to be 39 ± 4 min for control insects while the time for recovery for the *elav*; *SNCA*^*E46K*^*/+* flies was 54 ± 2 min. These data indicate lower ethanol resistance in *α*-synuclein transgenic flies (^*∗*^*p* < 0.05, ^*∗∗*^*p* < 0.01, by ANOVA; *n* = 32).

Behavioural assays regarding ethanol sensitivity were prepared by the valuation of sensitivity 50% (ST50) and recovery 50% (RC50) scores in the transgenic flies. The subsequent results are represented in Figure 8. A perusal of the figure indicates higher sensitivity and lower resistance to ethanol vapour among *E46K α*-synuclein expressing flies that was evident by lower ST50 and higher RC50 rates compared to the control genotype.

**Figure 9:**
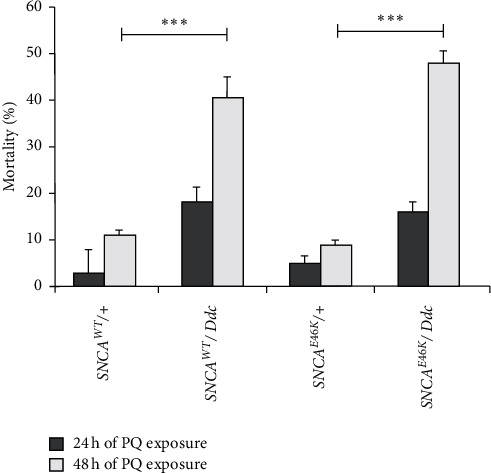
*SNCA* ^*E46K*^ flies are more sensitive to PQ toxicity. The mortality induced by PQ in flies exposed to the neurotoxin for 48 h was significantly high in *SNCA*-expressing transgenic insects when compared to control genotypes: *SNCA*^*WT*^*/+* and *SNCA*^*E46K*^*/+* (^*∗∗∗*^*p* < 0.001; by t-test *n* = 32). The average mortality of 46% is observed for *SNCA*^*WT*^*/Ddc* following 48 h PQ treatment while 49% for the *SNCA*^*E46K*^*/Ddc* genotype.

PQ neurotoxicity was evaluated by mortality percentage following 24 h and 48 h exposure of flies to 15 mM PQ (Figure 9). Human *SNCA* overexpression caused a high rate of mortality in the transgenic insects exposed to 48 h of PQ administration. The death rate was 18% higher in *E46K* when compared to *SNCA*^*WT*^.

Biochemical assessment of enzyme activities and OS markers levels are compiled in Table 1. The results revealed that *E46K α*-synuclein gene overexpression induces decreased catalase and superoxide dismutase.

Catalase and superoxide dismutase activities were measured as 4.4 ± 0.53 *μ*M·H_2_O_2_/min/mg protein and 0.47 ± 0.03 unit/mg protein, respectively, in transgenic flies and 6.5 ± 0.27 *μ*M·H_2_O_2_/min/mg protein and 0.62 ± 0.04 unit/mg protein, respectively, in control ones (*p* < 0.01, *n* = 9). Lipid peroxidation and ROS levels were significantly higher in *SNCA*^*E46K*^ model flies than control ones. Lipid peroxidation and ROS levels in transgenic flies were measured as 6.8 ± 0.26 nM MDA/mg protein and 895 ± 21.6 nM DCF/mg protein, respectively. The results revealed significant depletion in glutathione (GSH) level (12.8 ± 1.2 *μ*g GSH/mg protein) in PD model flies as compared to controls (18.5 ± 1.34 *μ*g GSH/mg protein; *p* < 0.01, *n* = 9).

## 4. Discussion

The mutations in the *SNCA* gene are rare, and the penetrance, pathobiology, and biochemistry of mutations remain to be fully investigated [7]. Genetic manipulation methods such as making transgenic *Drosophila* are valuable tools to assess such genetic malfunctions. In this study, genetic manipulation was achieved by overexpression of selected human gene under the control of an enhancer sequence, UAS (upstream activating sequence) in binary combination with *Gal4* as a transcription factor that can specifically bind to UAS and restrict the expression of the gene of interest both temporally as well as spatially [22]. Therefore, phenotypic consequences mediated by GAL4/UAS can be directly related to a distinct genetic manipulation. In this view, in the present study, transgenic flies bearing *E46K* mutant form of human *SNCA* gene were constructed, where *Drosophila* CNS neurons were targeted for *α*-synuclein gene overexpression and consequent effects on longevity, movement, ethanol tolerance, PQ-induced mortality, and cellular antioxidant defence system, *α*-synuclein were evaluated to study the neurotoxicity of the transgene.

A fly PD-like phenotype in *α*-synuclein transgenics was detected after day 10–12. Assessment of stable age-related molecular modification occurring prior to the beginning of neurodegeneration could reveal the causal biochemical events in the pathology of PD [18, 43]. *SNCA*^*E46K*^ transgenic flies used in the present study demonstrates the most significant behavioural defects in their climbing capability as compared to the control ones. Defective climbing activity is a well-characterized phenotype in all PD model flies [18, 44]. Since GAL4/UAS tool enables the tissue-specific expression of the human gene in defined developmental time, the observed locomotion impairment is due to neuronal demise in the fly brain but not muscle weakness.

The results of our assessments in *Drosophila* verified the association of *α*-synuclein overexpression with striking movement defects as characterized by negative geotaxis assay. Our results are in agreement with the results of other studies on the locomotor function of other *α*-synuclein transgenic animals [45, 46].

The decline in climbing ability, shortened longevity, and dopaminergic neuron loss in *E46K*-expressing flies are our findings that are in line with the results of Mohite et al. [19] on *Drosophila* models of PD overexpressing familial *α*-synuclein mutations. Ommatidial degeneration and locomotor deficits are reported by Sakai et al. [20] where they generated transgenic flies for *E46K α*-synuclein and other known mutations. These are the same as our study observations. In addition, the *E46K* variant was reported to be more resistant to degradation and thus had the highest level of expression when compared to other mutant variants [20]. This can support our hypothesis that *E46K* accumulation can cause OS as illustrated by Ingelsson [47].

The role of OS in the etiology of neurological diseases has been implicated in humans and most models of the disease [48]. OS is thought to be a series of pivotal biochemical events that may cause the onset of PD, *α*-synuclein aggregation, and dopaminergic neurons degeneration [49]. Intracellular overexpression of *α*-synuclein and the consequent ROS is involved in plasma membrane damage, mitochondrial dysfunction, and decline in glutathione level, all of which make the brain susceptible to oxidative damage [50].

Results of our study illustrated significant elevation of ROS and LPO levels and marked depletion of glutathione level in *α*-synuclein-expressing transgenic flies. Our findings are in line with those of other studies demonstrating higher levels of ROS as well as LPO in the PD model of *Drosophil*a [44, 45]. Interestingly, our results demonstrated diminished activity of CAT and SOD enzymes in *E46K α*-synuclein-expressing flies, as was reported in DJ-1 mutant flies [51] and *SNCA A30P* flies [34].

The *α*-synuclein-expressing transgenic flies demonstrate elevated sensitivity to neurotoxins in comparison to wild-type strains [52]. PQ, one of the most commonly used herbicides, can specifically affect dopaminergic neurons and lead to the development of movement disorders similar to PD symptoms [53]. Neurotoxicity of PQ could originate from its interaction mitochondria and inhibition of complex I leading to excess ROS generation and cell death [35]. Oxidative metabolism of ethanol persuades ROS production and OS. Ethanol exposure causes membrane lipid peroxidation [54], depletion of glutathione, and formation of protein carbonyl that serve as markers of OS in the brain [55]. Several dopaminergic neurons are engaged in ethanol-related impairments in mammalian CNS as well as in *Drosophila* [56]. It has been reported in our previous publication that *A30P* and *A53T α*-synuclein transgenic *Drosophila* have lower ethanol tolerance [29].

These findings support the idea that the production of free radicals and OS can heighten *α*-synuclein toxicity. Exposing *α*-synuclein PD model transgenic flies to neurotoxins such as PQ and ethanol incorporates both genetic and environmental factors and makes an ideal combined genetic-toxin in vivo model to study the interaction of pathogenic pathways that underlie the progression of PD.

Human *SNCA* has no *Drosophila* ortholog, and therefore, identification and characterization of gene networks and *α*-synuclein modifier genes are time-consuming and hard procedures. However, the present study provided powerful evidence for the contribution of *α*-synuclein to oxidative damage in the onset of PD symptoms in *SNCA*^*E46K*^ transgenic flies. This is similar to other *SNCA* mutations, that is, neurodegenerative, biochemical, and morphological changes. This study shows that this PD fly model provides a useful tool to make systematic and comparative assessments possible, to targeting efficient disease management strategies, prior to the start of its visible symptoms.

## Figures and Tables

**Table 1:** Overexpression of human *SNCA* leads to oxidative stress.

Genotype	CAT	SOD	GSH	LPO	ROS
UAS-SNCA^WT^/+	6.7 ± 0.32	0.58 ± 0.01	19.2 ± 2.03	6.1 ± 0.24	689 ± 25.6
*e*lav/+; SNCA^WT^/+	4.5 ± 0.24	0.49 ± 0.02	11.5 ± 1.50	7.0 ± 0.85	844 ± 11.3
*p* value	*p* < 0.01	*p* < 0.01	*p* < 0.01	*p* < 0.01	*p* < 0.01
UAS-SNCA^E46K^/+	6.5 ± 0.27	0.62 ± 0.04	18.5 ± 1.34	5.0 ± 0.47	700 ± 33.1
*e*lav/+; SNCA^E46K^/+	4.4 ± 0.53	0.47 ± 0.03	12.8 ± 1.02	6.8 ± 0.26	895 ± 21.6
*p* value	*p* < 0.01	*p* < 0.01	*p* < 0.01	*p* < 0.01	*p* < 0.01

Endogenous antioxidant enzyme activity and levels of oxidative markers are noticeably distorted and in *SNCA* transgenic PD model flies. Data represent mean ± SE of each biochemical marker. An independent sample *t*-test was applied, and corresponding *p* values indicate significant differences between tested genotypes. A total of 9 samples were analyzed in each genotype (*n* = 9). CAT = catalase-specific activity (*μ*M H_2_O_2_/min/mg protein); SOD = superoxide-dismutase-specific activity (units/mg protein); GSH = glutathione levels (*μ*g GSH/mg protein); LPO = lipid peroxidation levels (nM MDA/mg protein); ROS = reactive oxygen species levels (*μ*M DCF/mg protein).

## Data Availability

All the data are included within this article.
